# The Challenges for Manufacturers of the Increased Clinical Evaluation in the European Medical Device Regulations: A Quantitative Study

**DOI:** 10.1007/s43441-023-00527-z

**Published:** 2023-05-17

**Authors:** Breda Kearney, Olivia McDermott

**Affiliations:** College of Science & Engineering, University of Galway, Galway, Ireland

**Keywords:** MedTech, Medical device, European Medical device regulations, Clinical trials, Clinical evaluation

## Abstract

The European Union Medical Device Regulations 2017/745 entered into force on May 2021 with changes related to strengthening the clinical evaluation requirements, particularly for high-risk devices. This study investigates how the increased requirements on medical device manufacturers in relation to how clinical evaluation will challenge manufacturers. A quantitative survey study was utilized with responses from 68 senior or functional area subject matter experts working in medical device manufacturing Regulatory or Quality roles. The findings from the study demonstrated that the highest source of reactive Post-Market Surveillance data was customer complaints and proactive data were Post-Market Clinical Follow-Up. In contrast, the top 3 sources for generating clinical evaluation data for legacy devices under the new Medical Device Regulations were Post-Market Surveillance data, Scientific literature reviews, and Post-Market Clinical Follow-Up studies. Manufacturers’ biggest challenge under the new Medical Device Regulations is determining the amount of data needed to generate sufficient clinical evidence, while over 60% of high-risk device manufacturers have outsourced the writing of their clinical evaluation reports. Manufacturers also reported a high investment in clinical evaluation training and highlighted inconsistencies in the requirements for clinical data by different notified bodies. These challenges may lead to a potential shortage of certain medical devices in the E.U. and a delay in access to new devices, negatively impacting patient quality of life (1). This study provides a unique insight into the challenges currently experienced by medical device manufacturers as they transition to the MDR clinical evaluation requirements and the subsequent impact on the continued availability of medical devices in the E.U.

## Introduction

Medical devices are diverse and can range from simple devices such as bandages to more complex devices, such as implantable devices, stents, and smart devices, which rely on software and the Internet for enhanced connectivity [1, 2] . Medical devices and pharmaceuticals are among the most highly regulated industry sectors [3]. Regardless of the type of device or its intended use, a medical device must not lead to unanticipated side effects or serious health complications for the end user [4].

Before a medical device is placed on the European market, the device manufacturer must demonstrate to their notified body (N.B.) that the device is safe, has efficacy, performs as intended, and does not cause unexpected harm to a patient during normal use and functions as expected [5]. One of the methods by which manufacturers demonstrate safety is by generating a clinical evaluation report (CER) containing documented clinical evidence to support their statements regarding device safety and performance. This is a critical document submitted to their notified body to support their marketing authorization application to obtain a C.E. [6]. Once the N.B. accesses the CER and the other components of the regulatory submission and is satisfied that the device is safe for use and performs as intended, the manufacturer may market the device. Regulatory Strategy is based on having design, manufacturing, and market vigilance control and procedures as part of the device product lifecycle [7]. Through carrying out market vigilance and post-market surveillance (PMS) throughout the lifetime of the medical device, the manufacturer is expected to update their CER and other relevant technical documentation [8].

Before May 2021, European medical devices were regulated under the MDD/AIMDD (medical device directive and active medical device directive) [9]. However, following several scandals related to medical devices and public outcry, the E.U. recognized that the directives were inadequate for regulating medical devices and needed improvement; thus, the directives were repealed [10]. The new EU-MDR 2017/745 (MDR) replaced the directives, which came into force on May 27th, 2021 [11]. The new MDR is intended to provide stronger regulatory oversight and rigor throughout the medical device lifecycle to ensure patient safety [12].

Many commentators have discussed how the stricter clinical evaluation requirements required under the new MDR will present enormous challenges to manufacturers [13]. These challenges are specifically related to the amount and type of clinical data required to demonstrate device safety and performance and to support an acceptable benefit–risk ratio, which increases compared to the MDD/AIMDD. In addition, the relevant European competent authority can also scrutinize the N.B. assessment, so manufacturers may experience delays in product reviews and increased requirements to deliver clinical data quality and quantity [11, 14].

Before the MDR, manufacturers may have relied on demonstrating equivalence to an already marketed device to support their regulatory approval submission, thus reducing pressure to generate clinical data [5]. However, the MDR introduces much stricter requirements for demonstrating equivalence. If the device manufacturer cannot obtain sufficient data to demonstrate conformity to the new requirement, they cannot use an equivalence case and must produce clinical data to support their C.E. marking application [15].

Manufacturers, thus, must carry out clinical investigations to generate appropriate clinical data for certain high-risk and legacy devices that can no longer rely on demonstrating equivalence [12].

In some cases, the cost of generating the required clinical data will outweigh the potential return on investment and may result in devices being taken off the market and subsequent device shortages [16–18]. Thus the challenges faced by medical device manufacturers in meeting the strengthened clinical evaluation requirements under the MDR pose a potential risk to the healthcare supply chain. As a result, manufacturers may discontinue certain medical devices, remove them from the E.U. market, and reconsider seeking approval for more innovative medical devices, creating shortages in certain patient cohorts [11, 19].

The MDR came into force in May 2021 and with the deadline for completion of the transition in manufacturers’ minds, they are under pressure to either obtain C.E. marking under the MDR or have their devices removed from the E.U. market [6]. Furthermore, several studies report that compliance with the MDR’s clinical evaluation requirements poses a significant challenge for manufacturers [10, 13, 19]. However, to the author’s knowledge, no published studies with data identifying the barriers that the clinical evaluation poses to ensuring compliance with the new MDR exist. Thus, this research will provide new and original insight into the difficulties faced by the device industry in conforming to the enhanced clinical evaluation requirements. Within this context, this research project will investigate the following research questions (R.Q.s):

1. What challenges are manufacturers facing when generating an MDR-compliant Clinical Evaluation Report (CER) and a CER for legacy devices?
2. What are the sources for obtaining clinical evaluation data information?

Section “Background to this Study” will provide the background literature on the MDR and CER requirements. Then, Sect.  “Methodology” outlines the methodology and Sect.  “Results” outlines the results. Finally, the discussion and conclusion are outlined in Sects. “Results” and “Discussion”.

## Background to this Study

### The E.U. Medical Device Market Introduction & Requirements

Europe represents the second largest market globally, with a value of USD 140.07 billion in 2022 (Market Data Forecast, 2022). The two directives which governed Medical devices in Europe the Council Directive 93/42/EEC on Medical Devices (MDD) (1993) and Council Directive 90/385/EEC on Active Implantable Medical Devices (AIMDD) (1990). A number of highly publicized scandals related to medical devices in Europe occurred in recent years—the Poly Implant Prothese (PIP) breast implant scandal and the DePuy hip implant incident [20, 21] led to concern about the stringency of European regulations. Investigations of these scandals raised issues around the approval processes for medical devices and N.B. scrutiny and practices [22, 23]. Public and political pressure led to a review by the European Union and the creation of a new, more stringent MDR. While the MDR came into force on May 26, 2021, which means from then, new medical devices can only be placed on the market if they are C.E. marked under MDR. Post-May 2024, legacy devices already legally on the market can continue to be used until May 2025; the E.U. has announced an extension to this previous date [24].

The MDR is a more lengthy and comprehensive document than its predecessors (MDD 93/42/EEC—60 pages, AIMD 90/385/EEC—20 pages). Annex Chapter VI Article 61 on clinical evaluation contains the requirement to conform to the general safety and performance requirements (GSPRs), whereas Annex XV Chapter VI Article 62 requires a clinical investigation to be conducted [12]. Clinical evaluation requirements have been strengthened for high-risk and implantable devices, and evidence of the safety and performance of the device must be obtained from clinical investigations. The CER must be updated for all device types using PMS data and aligned to the risk management system [12].

The new MDR also introduces a clinical evaluation consultation procedure and scrutiny procedures. These new procedures related to clinical evaluation consultation and scrutiny have been introduced in the MDR. These procedures are aiming to improve oversight of the notified bodies by the relevant county-specific competent authority by ensuring more inspection around the N.B.’s evaluation of the manufacturer’s CER. For certain high-risk devices, the notified bodies’ clinical evaluation assessment report must be reviewed by expert panels [25].

Manufacturers have traditionally viewed Europe as the market to launch their products, followed by the U.S. market at a later stage. This has occurred partly due to less stringent demands for pre-market clinical data in Europe than in the U.S.A. [26, 27]. The requirement for less clinical data has led to some high-risk medical devices receiving market approval in Europe several years earlier than approval via the FDA Pre-Market Authorisation (PMA) [26, 28]. However, The FDA has stated that several high-risk medical devices were approved for use in Europe before the U.S.A. based on reduced clinical data requirements, which were later withdrawn from the European market due to safety issues [29].

To place a medical device on the European market and gain the C.E. mark, the device’s technical documentation, including the CER, is submitted to the N.B. to demonstrate device safety and performance [12]. If the N.B. assesses the technical documentation and concludes that the device is safe and performs as intended in accordance with the manufacturer’s intended use statement, the C.E. mark is placed on the device.

### Sources of Clinical Evaluation Data

Peer-reviewed scientific literature and clinical investigations are one of the main sources for clinical evaluation. The methodology by which the literature review is carried out must be documented, e.g., search engine used and conclusions [6]. If there are gaps in data, they must be supplemented with other clinical sources. A clinical investigation refers to a formal assessment of the devices safety and performance in one or more human subjects. These clinical investigations are usually conducted under the requirements of ISO 14155, the standard for clinical investigation of medical devices for human subjects—Good clinical practice. Clinical investigations are the most common source of clinical data [15]. Under the MDD and AIMDD manufacturers could claim that their device is equivalent to another device. This claim that the device is at least as safe for use and has a similar level of performance to a CE-marked device currently on the market could enable bypassing the requirement for clinical investigations.

#### Clinical Evaluation – Post-Market Clinical Data Sources

The MDR introduces more prescriptive requirements for PMS and PMCF. Peer-reviewed scientific articles can also describe the real-world clinical experience on the use of the device in support or otherwise of device safety, performance, and clinical benefits and can also be used to identify the need for modification or changes to the original design of the medical device. The outputs of the PMS system are also used to update the risk management file and clinical evaluation.

Clinical data generated through PMCF is also used to (1) support the device benefit–risk ratio, (2) support the claimed lifetime, and (3) verify that the intended purpose statement is correct [6].

PMCF is required for all devices unless a valid justification can be provided. PMCF data generated under the directives may not be sufficient to demonstrate compliance with the EU-MDR, particularly for devices that can no longer claim equivalence. Therefore, manufacturers of legacy devices need to consider the role of PMCF activities as part of their EU-MDR transition strategy under the Medical Device Coordination Group (MDCG) MDCG 2020-6 [1].

## Methodology

A Quantitative Study utilizing a survey was utilized to answer the research questions. Given the complexity of the research topic under investigation and to ensure the generation of reliable research data from which valid conclusions can be drawn, it is important that the survey data is derived from (1) individuals with appropriate knowledge of the research topic [30] and (2) includes representation of both large and SME companies. Considering that most of the respondents work directly in quality and regulatory affairs, of which over 60% are at a managerial level or higher, it is concluded that the respondents have the required knowledge and insight to answer the survey questions accurately. 80 MedTech professionals were invited to participate in the online survey, most of whom work directly in senior roles in quality, regulatory and/or clinical affairs. As a result, 68 completed the survey providing a response rate of 85%. The survey questions are outlined in Table 1. All questions arose from literature-related themes related to Clinical Evaluation and MDR.

**Table 1:** Survey Questions.

Non-Demographic Survey Questions	
What information sources do you use to guide your MDR clinical evaluation strategy? Please tick all that are appropriate?	a) In-house knowledge and expertise, b) External experts/consultants, c) MDCG Guidance Documents, d) MEDEV 2.7.1 Rev 4, e) External Training, f) Notified Body publication/events/updates/webinars, g) IMDRF MDCE, h) MedTech Europe.
For legacy devices, what are the main data source(s) that your company will use to generate a clinical evaluation under MDR?	a) New Clinical investigation/trials b) PMCF Studies c) PMCF Survey d) PMS Data e) Scientific literature reviews f) Device registries g) Data from RWE h) Other
What reactive data source(s) do you use for PMS?	a) Customer Complaints b) Analysis of serious incidents -FSCA/Field Safety Notices c) Maintenance & Service Notices d) Reviewing PSUR’s e) Electronic health/Medical Records f) Healthcare Claims databases g) Data generated by digital health technologies h) Literature reviews
What proactive data source(s) do you use for PMS?	a)Feedback from focus groups b) PMCF Surveys (patient/end-user) c) Monitoring Recalls and/or other safety issues associated with devices (e.g., MAUDE) d) Attendance at conferences/events e) PMCF studies f) Manufactured sponsored device tracking/implant registries g) Regional or national device registries and/or databases
For scientific literature reviews, what databases are used to source relevant articles?	a) Cochrane b) PubMed/Medline c) EMBASE d) Web of Science e) Science Direct f) Google Scholar g) I don’t know
Select the 3 main challenges manufacturers face when conducting a scientific literature review?	a) Establishing a reproducible literature review protocol b) Defining inclusion and exclusion criteria c) Development of an in-article appraisal strategy d) Gaining access to databases e) Developing appropriate search terms f) Ensuring cover of similar and/or equivalent devices g) Don’t know
Does your company outsource the generation of your clinical evaluation report (CER)?	a)Yes, for all devices b) Yes, but only for certain high-risk devices c) Yes, but only since transitioning to EU-MDR d) No, we generate our CER in-house
If your company outsources your CER, what is the rationale for outsourcing?	a) Lack of external expertise b) More cost-effective c) We do not have the time and resources to generate and maintain the required documentation.
Does your regulatory team use any of the following documents?	List of 7 guidance documents and 4 templates/application forms provided.
Compared to the MDD/AIMD, what are the 3 main challenges faced by manufacturers when generating a clinical evaluation under MDR?	a) Identify a clinical evaluation pathway. b) Amount of data needed to generate sufficient clinical evidence c) Time of resolution of clinical queries from appropriate notified bodies

50% of respondents worked for a large global multinational medical device company (> 250 employees), 7% worked for large Irish-based companies (> 250 employees), and approx. 43% worked for a SME company (> 10 − 250 employees).

The demographics of the respondents in terms of functional level and functional areas worked are outlined in Table 2. Over 80% of the respondents worked in senior management/director roles across Regulatory Affairs, Quality Assurance, Clinical, and the QARA function. Importantly, the 68 respondents represented 68 different organizations and only one relevant functional stakeholder was invited from each organization. In that way, the study has a strong representative sample across the MedTech industry.

**Table 2:** Respondent Profiles.

Qty of Respondents	Function	Level
35%	R.A	Senior Mgmt
26%	Q.A	Senior Mgmt
15%	Clinical	Senior Mgmt
4%	QARA	Senior Mgmt
10%	R.A	Middle Mgmt/Specialist
1%	QARA	Middle Mgmt/Specialist
7%	Other	Engineer

## Results

Considering that the clinical evaluation requirements are stricter for high-risk medical devices, the results were analyzed by comparison of the responses from participants assigned to groups based on the highest-risk class in their product portfolio. Therefore, the final survey population had 69% assigned to the 'High-Risk Device Group’ and the remaining 31% were assigned to the ‘Medium-Risk Device Group.’ In addition, where there were comparisons to be drawn, the differences in SMEs versus L.E.s were also analyzed.

### Information Sources for Clinical Evaluation

Developing an effective and regulatory-acceptable clinical evaluation strategy relies on understanding the expectations of MDR. Medical device professionals from various sources can obtain knowledge and information on the requirements for clinical evaluation.

**Figure 1:**
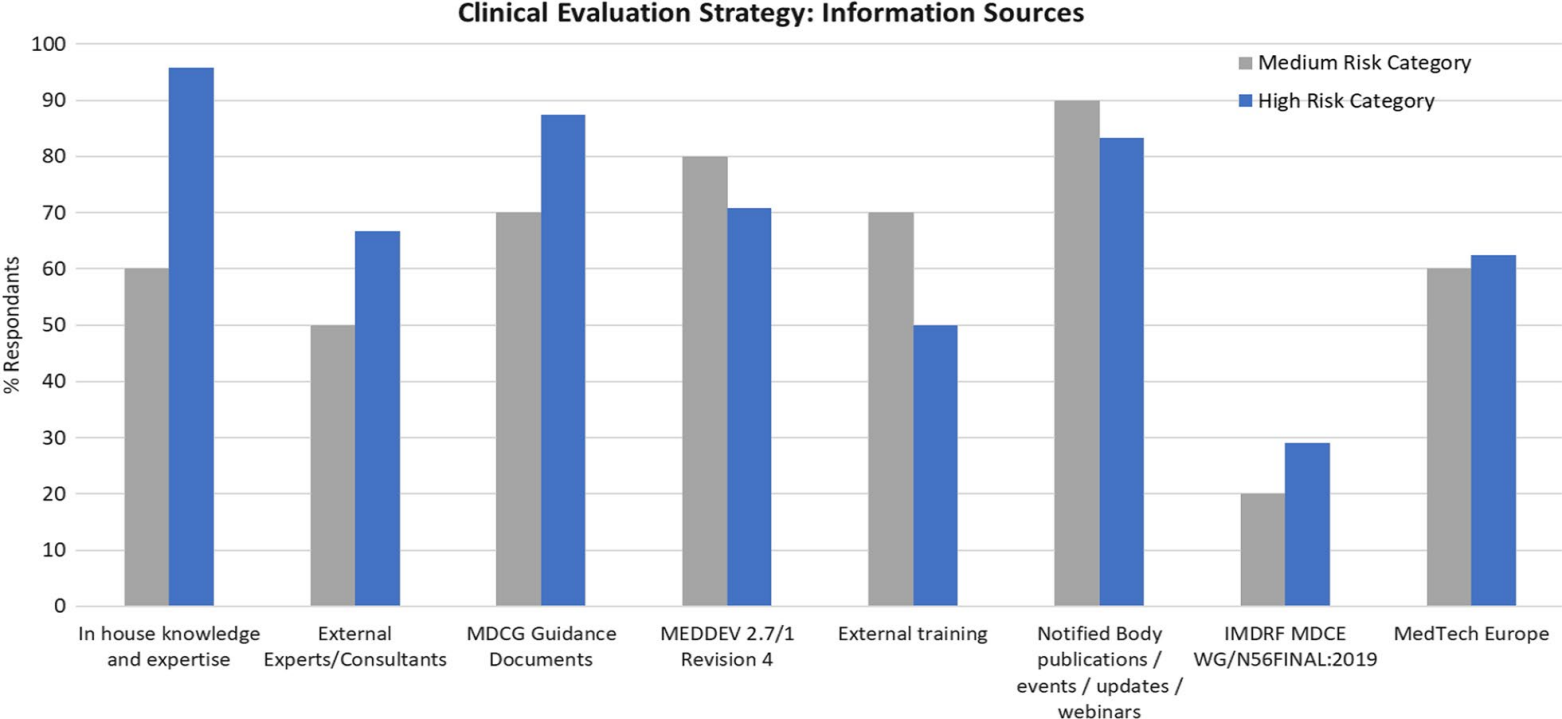
Comparison of the information sources used by the survey respondents (expressed as a % of the total population to guide their clinical evaluation strategy.

In this study, approx. 96% of respondents in the high-risk category relied predominantly on obtaining information by accessing ‘in-house knowledge and expertise,’ followed by referring to MDCG guidance documents (approx. 88% of respondents) which are freely available on publication from the European Commission. Approx. 83% of respondents use notified bodies, placing this category as the third most popular information source (Fig. 1). It is interesting to observe that only 50% of respondents in the high-risk category identified external training as an information source, as given the complexity of the Regulation, there is an expectation that this value would be higher. A possible explanation could be that manufacturers of high-risk medical devices initially relied on external training in preparation for their transition to MDR. However, this reliance may decline as the knowledge has been transferred in-house, coupled with increased availability of MDCG guidance documents. For the medium-risk category group, the top 3 information sources were notified bodies (90%), MEDDEV 2/71/1 (80%), and in joint third place, external Training and MDCG guidance documents (70%).

Despite being referenced by MDCG 2020–6 as an information source for developing criteria for appraisal of clinical data, it was surprising that IMDRF MDCE WG/N56 FINAL Clinical Evaluation was the least referenced source of information for both groups. However, this could be explained by greater familiarity with and use of MEDDEV 2.7/1. As there were 57% L.E.s versus 43% SMEs in this study, there were few differences of note between the information sources utilized by L.E.s versus SMEs. However, L.E.s had more in-house knowledge due to larger resources, so they generally did not have to use external consultants as SMEs do, according to the respondents (60% of SMEs used external consultants versus 40% of L.E.s).

### Legacy Devices Clinical Evaluation

**Figure 2:**
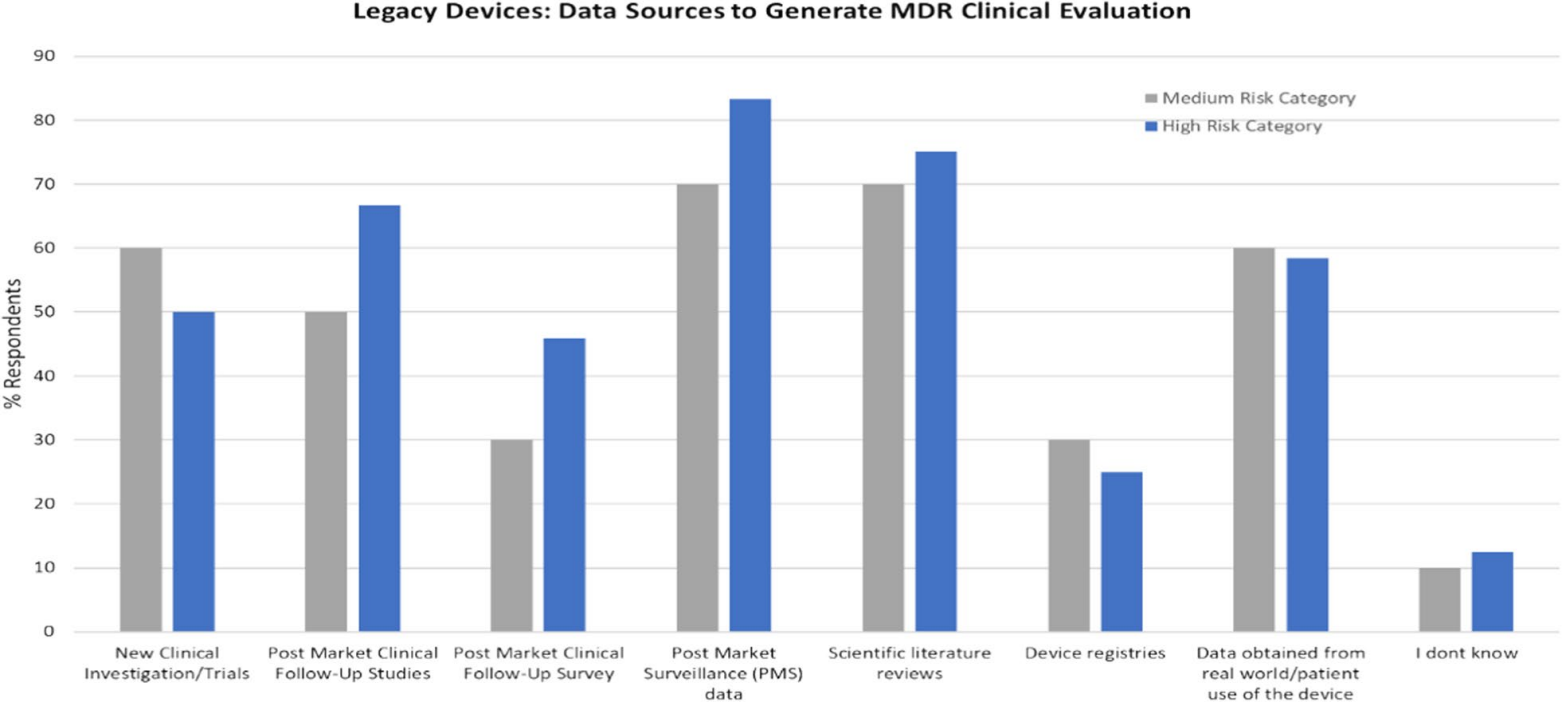
Comparison of the data sources used by the survey respondents (expressed as a % of the total population) to develop their clinical evaluation for legacy devices.

For legacy devices transitioning to MDR, planning to address gaps in clinical data is an essential element of the manufacturer’s clinical evaluation strategy. Depending on the extent of the data gap, new or supplementary clinical data can be obtained from various sources, as shown in Fig. 2.

In the case of high-risk medical devices, the top 3 sources are PMS data (approx. 83%), Scientific literature reviews (75% of respondents), and PMCF studies (approx. 67%). Similarly, for medium-risk devices, PMS data and scientific literature reviews represented the two main clinical data sources (70%). This finding signifies the importance of implementing an effective PMS system and having access to sufficient and competent resources for conducting an effective scientific literature review.

60% of respondents of medium-risk legacy devices and 50% of respondents of high-risk legacy devices indicate their intention to conduct a new clinical investigation. The intention to use this data source suggests that, perhaps due to the reliance on demonstrating equivalence under the MDD, the original clinical data used for C.E. marking is insufficient for demonstrating device safety and performance under MDR. There were no real differences between SMEs versus L.E.s regarding the sources to generate their CER, as all organization sizes used all sources.

### PMS Data Sources

Data collected via the manufacturer’s PMS system is a key input to the device’s clinical evaluation. Given the range of data sources available for obtaining reactive and proactive PMS data, it is interesting to determine the top 3 data sources and if there are any differences depending on the risk level of the device.

For both the high-risk and medium-risk groups, the main source of reactive data was customer complaints (approx. 88% of respondents versus 100%). Analysis of vigilance data, including serious incidents, FSCAs, and FSNs, was the second most widely used data source for high risk versus medium risk (approx. 83% respondents versus 70%). Finally, the third most used reactive data source was literature reviews (75% of respondents versus 50%). The least utilized sources were EHRs, maintenance and service reports, data from digital health technologies and claims databases. Given that the aforementioned sources’ applicability depends on the type of device, it is not surprising that the respondents less frequently used these sources.

The combined results of PMCF surveys and PMCF studies represent the second most widely used sources of proactive data for both groups. However, given the more stringent clinical data requirements for high-risk devices, it is perhaps not surprising that the % of respondents conducting PMCF studies is lower than 40% for respondents in the medium-risk group compared to approx.—58% for the high-risk group.

### MDD v MDR CER Challenges

This question aimed to identify the top 3 challenges manufacturers face when developing an MDR-compliant CER.

**Figure 3:**
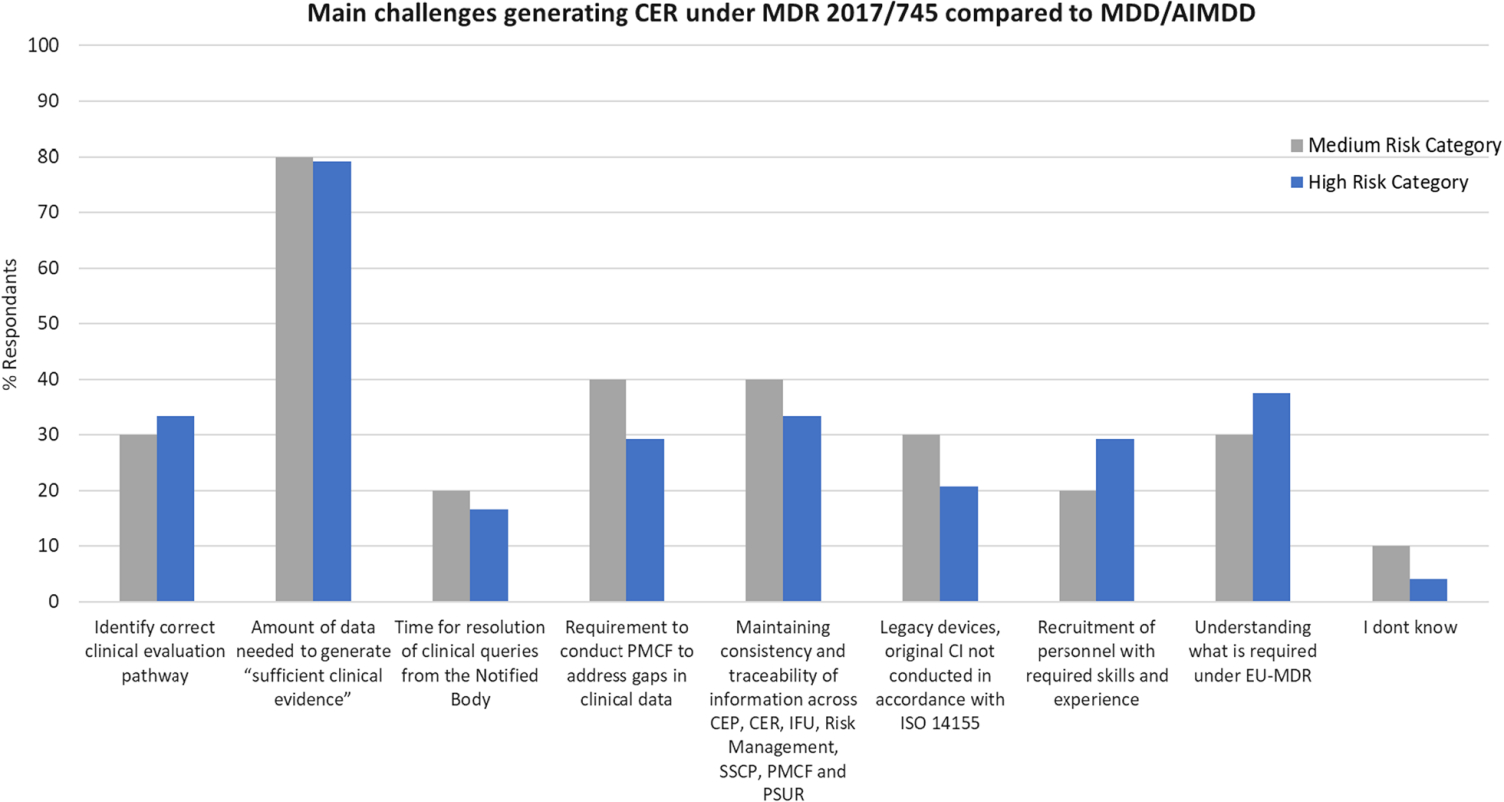
Comparison of the main challenges encountered by the study population (expressed as a % of the total population) when developing a CER under EU-MDR compared to the directives.

The challenges listed in Fig. 3 were identified based on the findings from the literature review stage of this study. The results demonstrate that manufacturers’ biggest challenge, irrespective of device risk class, is determining the “*Amount of data needed to generate sufficient clinical evidence*.”

While a large amount of data are always a challenge in any new regulation or regulatory change, it has been reiterated repeatedly within the literature that this new clinical data requirement, which had never been present at that level previously, is leading to problems for MedTech organizations [10, 16, 18, 22, 27]. The findings from the survey back this up. Even more worryingly, the data quantities required have led to certain devices being taken off the market and to medical device shortages in Europe [18, 24, 31]. There is little difference for the medium-risk group between the remaining listed challenges, with the % of respondents per challenge at about 30–40%. Similarly, for the high-risk group, there was little separation between the % of total respondents for the remaining challenges. It was promising to note that in this study, < 20% of respondents reported that delays in clinical query resolution from the notified body posed a challenge. This finding suggests that queries are readily understood and resolved when generated or the number of queries generated was not deemed problematic for the manufacturer.

### Questions related to the Outsourcing of CER

Studies have suggested that under MDR, there has been an increase in outsourcing of the device’s clinical evaluation, irrespective of the company size (large v SME) or device risk classification. To examine this hypothesis, a question was asked to determine the extent of outsourcing and the rationale for outsourcing, where applicable.

**Figure 4:**
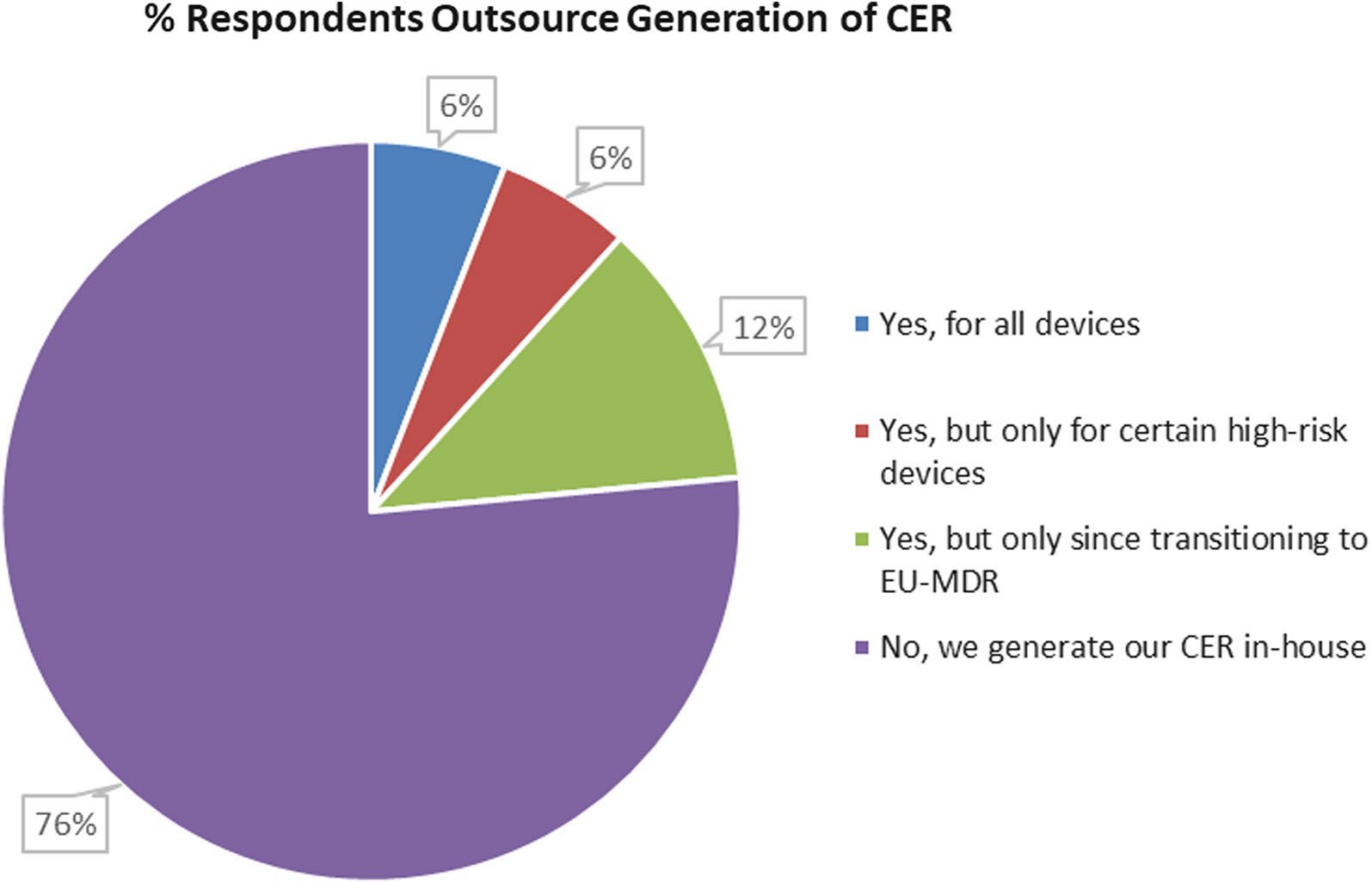
The extent to which the survey respondents (expressed as a % of the total population) outsource the clinical evaluation report generation to an external expert.

In this study, as depicted in Fig. 4, most of the respondents (76%) do not outsource the generation of the clinical evaluation report. Of the remaining respondents, 12% indicated that the CER was generated in-house before MDR was implemented. In addition, 6% indicate that they outsource the CER for all devices and 6% indicate that the CER is only outsourced for certain high-risk devices. When this data was analyzed of those who did not outsource, the majority were large enterprises (L.E.s) rather than SMEs.

**Figure 5:**
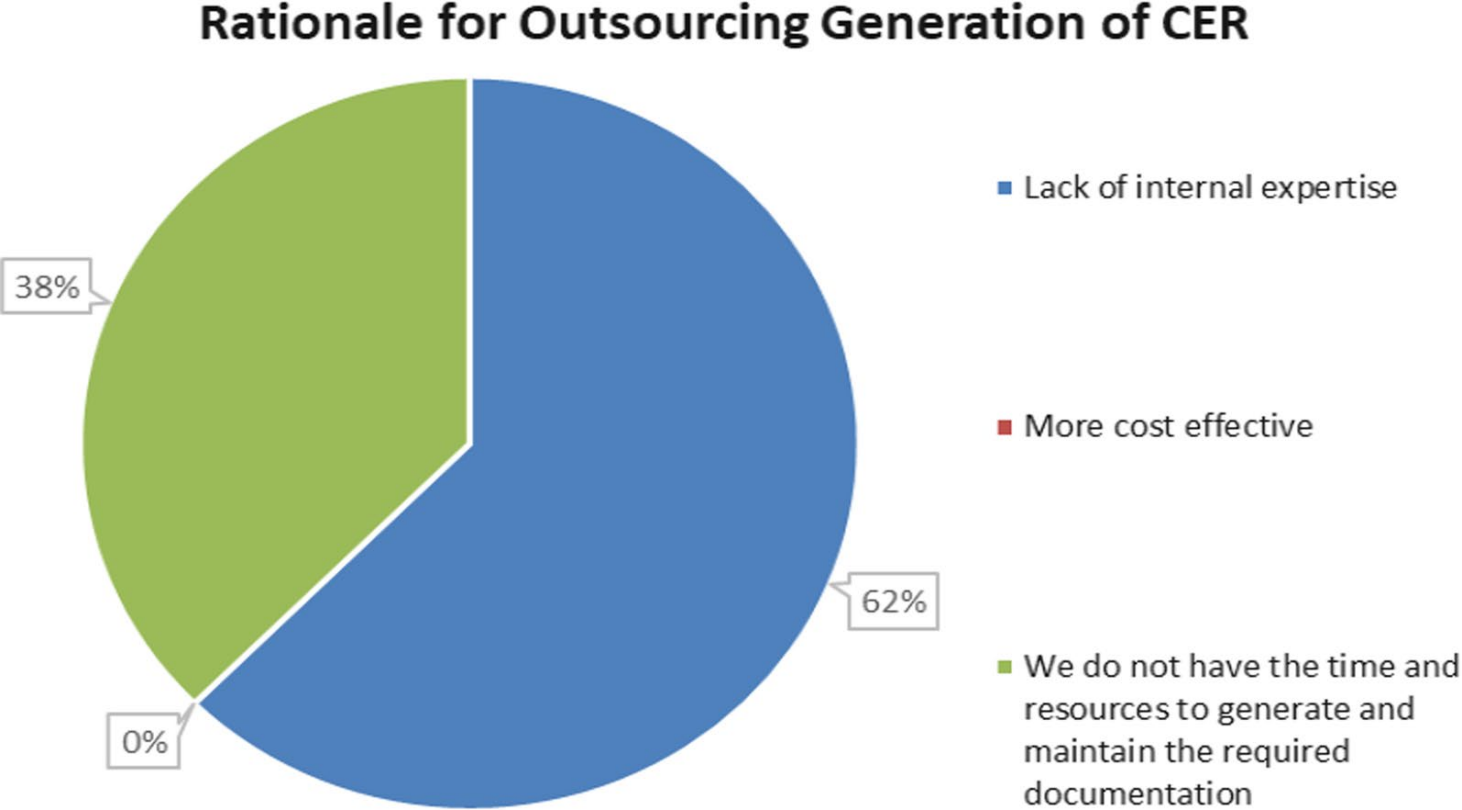
Comparison of the rationale for outsourcing generation of the clinical evaluation report (expressed as a % of the total population who responded that they outsource the CER in the previous question).

In the next survey question, the respondents were asked to explain their outsourcing rationale. The results are presented in Fig. 5. Although it was found that there was a low % of respondents who outsourced their CER, this question was asked to ascertain what types of organizations were outsourcing.

The resulting data show that a lack of internal expertise (62% of respondents) is the primary reason for outsourcing the clinical evaluation. Further data analysis was performed to determine if there was a trend in the extent of outsourcing between SMEs versus large medical device manufacturers. From the survey respondents who work for a SME company versus a large company, 50% of SMEs responded that they outsource their CER compared to 18% for large company respondents, and the primary rationale was attributed to a lack of internal expertise. Outsourcing has implications for resources, and SMEs tend to lack internal expertise compared to L.E.s. Notably, no respondents (0%) stated they outsourced because it was more cost-effective, indicating outsourcing was a necessary evil.

### Guidance Documents used by the Regulatory Team

This question aimed to determine what guidance documents the respondents’ regulatory teams use. The rationale for selecting the guidance documents listed in the survey is as follows. The E.U. Commission provides guidance to manufacturers on the implementation of MDR via the publication of MDCG guidance documents. In relation to clinical evaluation, 7 guidance documents and 4 templates/application forms are currently available. For the purposes of this study, three guidance documents which speak specifically to the generation of clinical data were selected: MDCG 2020-6 Guidance on sufficient *clinical evidence for legacy devices, MDCG 2020*-*5* Guidance on *clinical evaluation*—*Equivalence, MDCG 2021–6* Regulation (E.U.) 2017/745*—Questions & Answers* regarding *the clinical investigation.*

1. In the absence of guidance on using RWE in support of C.E. marking, the researcher aimed to determine the level of awareness among regulatory affairs professionals of FDA and NESTcc efforts to provide manufacturers with guidance on the generation of RWE, which is fit for regulatory use.
2. The E.U. commission provides guidance to manufacturers on implementing MDR via the publication of MDCG guidance documents. In relation to clinical evaluation, 7 guidance documents and 4 templates/application forms are currently available. For the purposes of this study, three guidance documents which speak specifically to the generation of clinical data were selected: MDCG 2020–6 Guidance on sufficient clinical evidence for legacy devices, MDCG 2020–5 Guidance on clinical evaluation—Equivalence, MDCG 2021–6 Regulation (E.U.) 2017/745–Questions & Answers regarding the clinical investigation. More than 60% of respondents reported that their regulatory teams use MDCG guidance documents. This finding is not surprising given that a previous question (Sect.  “Information Sources for Clinical Evaluation”) found that MDCG documents represent one of the main sources for obtaining information on MDR.

### Influence of MDR on Strategic Planning and Experience of Notified Body Clinical Data Review

**Figure 6:**
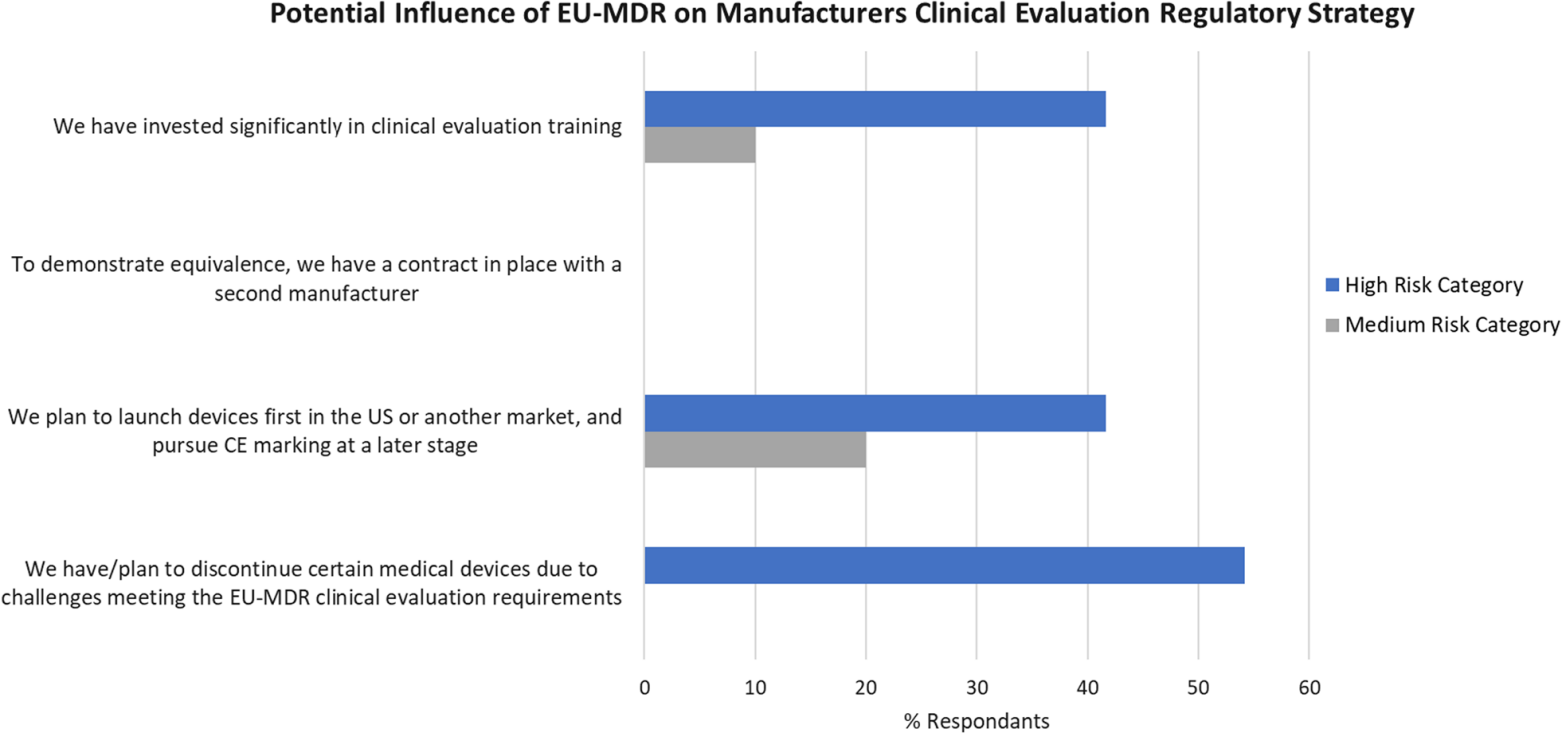
Potential influences on EU-MDR clinical evaluation regulatory strategy—comparison of % respondents who selected each listed statement.

As depicted in Fig. 6, approx. 42% of respondents in the high-risk device category responded that they had invested significantly in clinical evaluation training compared to just 10% of respondents in the medium-risk category. This finding is not surprising as the clinical evaluation for higher-risk devices is subject to greater scrutiny by the notified body and, for certain devices, by an expert panel. In addition, none of the respondents has a contract in place with a second manufacturer (more than likely a competitor) to gain access to the device’s technical documentation. A lower % of L.E.s had invested in clinical evaluation training than SMEs. This is attributed to SMEs’ lower resources and experts’ availability.

In recognition of the challenge manufacturers of high-risk devices faced in demonstrating compliance with the stricter requirements for clinical evaluation introduced under MDR, approx. 54% of respondents have or are planning to remove medical devices from the E.U. market. Whereas for the medium-risk group, no respondents indicated their intention to remove devices from the market. This finding correlates with the fact that the biggest challenge the respondents highlighted in generating the CER was the amount of data required, thus causing difficulties with keeping products certified and on the market [24].

It is also interesting to observe that 41.7% of respondents in the high-risk device group have made the strategic decision to launch devices in another market first and then pursue C.E. marking at a later stage, compared to 20% of respondents in the medium-risk device group. Serious challenges are being faced by manufacturers, particularly of high-risk devices, in demonstrating compliance with MDR.

**Figure 7:**
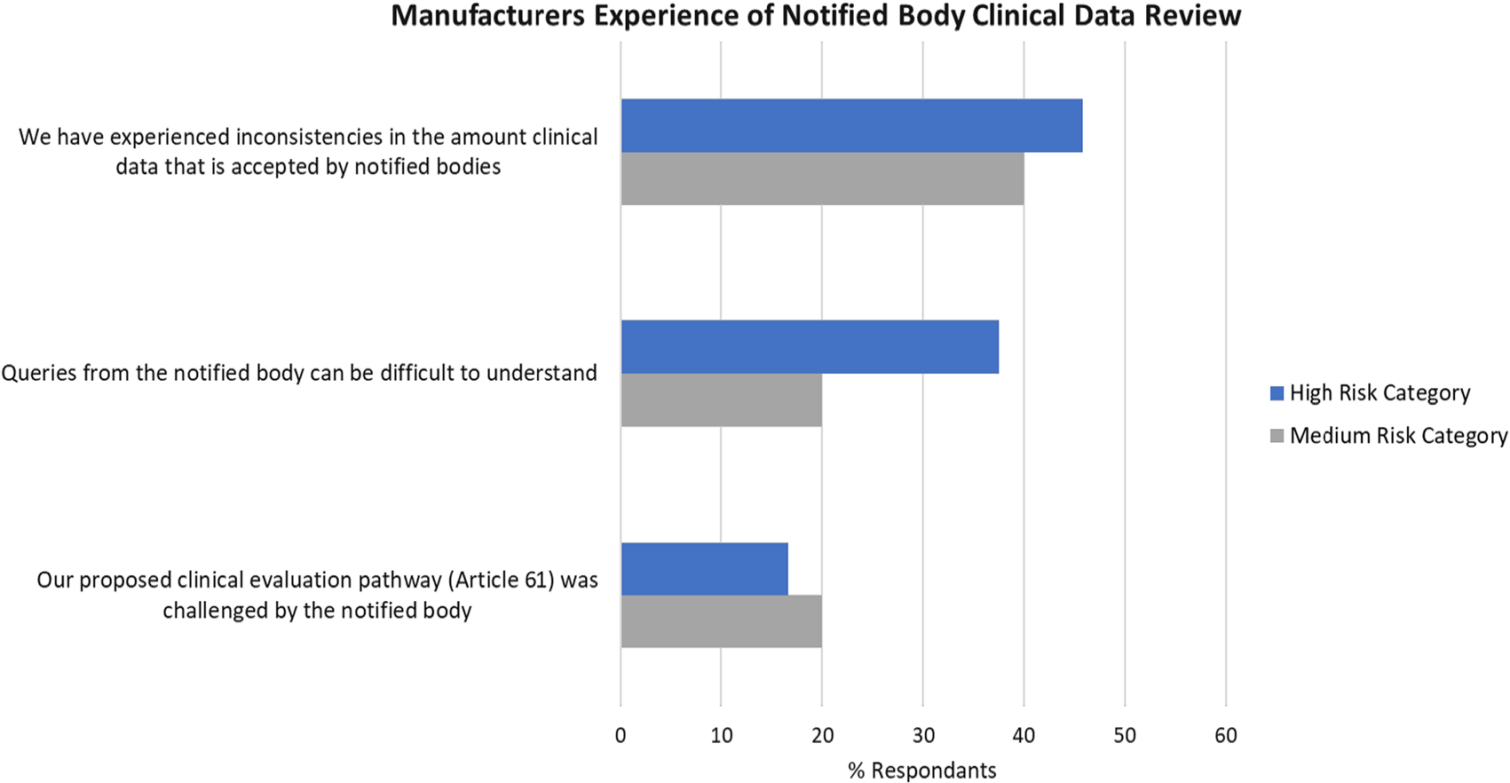
Experience of clinical data review by the notified body EU-MDR clinical—comparison of % respondents who selected each listed statement.

As depicted in Fig. 7, approx. 46% of respondents in the high-risk group and 40% in the medium-risk group have experienced inconsistencies in the amount of clinical data accepted by the different notified bodies. This finding supports the additional oversight introduced under MDR to ensure consistency in applying the requirements. However, without guidance on what constitutes sufficient clinical evidence, it is challenging for all stakeholders to consistently apply the clinical evaluation requirements. L.E.s and SMEs experienced the same challenges as each other in their notified body clinical review.

## Discussion

### Research Question 1

What challenges are manufacturers facing when generating a MDR-compliant Clinical Evaluation Report (CER) and a CER for legacy devices?

Several authors suggest that meeting the requirement to generate sufficient clinical evidence supporting C.E. marking may pose the greatest challenge for manufacturers [14]. The literature review also determined that in the absence of a cost-effective approach for generating the required clinical evidence, some manufacturers are expected to make the strategic decision not to pursue MDR CE marking for certain devices [11]. The data generated from this research project corroborate these findings, irrespective of the device’s risk classification or the organizational size, determining what constitutes sufficient clinical evidence represents the main challenge when generating an MDR-compliant CER.

The term-qualified assessment in the MDR implies that certain competencies are required to generate an MDR-compliant clinical evaluation and to identify potential data gaps that require remediation [15]. This is also supported by the finding that “understanding what is required under the MDR” is one of the top 3 challenges reported by the survey respondents when generating an MDR-compliant CER.

In this study, approx. 42% of respondents from manufacturers of high-risk devices report that their organizations have invested significantly in clinical evaluation training compared to just 10% for the medium-risk device group. This finding suggests that these manufacturers appreciate the difference in the clinical evaluation requirements under the MDR compared to the directives and that training is essential to understand the expectations for demonstrating compliance.

To aid in their understanding of the clinical evaluation requirements, manufacturers of all devices, irrespective of their risk classification, can refer to publicly available MDCG guidance documents, particularly MDCG 2020-13 and MDCG 2020-6 [15], whereas MDCG 2020-6 provides guidance on what constitutes sufficient clinical evidence for legacy devices. This study found that approx. 40% of medium-risk device manufacturers and approx. 45% of high-risk devices have experienced inconsistencies in the amount of clinical data accepted by notified bodies. In the absence of clarification from the European Commission regarding their expectations on what constitutes sufficient clinical evidence for specific types/groups of devices, confusion regarding how much clinical data is required is likely to continue resulting from inconsistent demands being placed on the notified bodies from each individual competent authority, and these, in turn, being passed down to the manufacturer.

Given that the time taken for the notified body to complete their assessment of the clinical evaluation report is already expected to increase under the MDR [14], manufacturers must ensure that their supporting documentation complies with the expectations of the MDR and is fit for regulatory submission. The results of this research point toward a knowledge gap in relation to the requirements of the MDR. Thus, it is unsurprising that “*Maintaining consistency and traceability of information*” was the second highest challenge reported by the survey respondents.

To address some of the challenges they are facing, manufacturers may decide to outsource the generation of their device’s clinical evaluation to an external consultant. Interestingly in this study, the extent of outsourcing was low with most respondents (approx. 74%) generating their CER in-house. Furthermore, outsourcing the CER was more common in SMEs than in larger organizations, primarily due to a lack of internal expertise. The need to outsource the generation of their CER to external clinical evaluation consultants due to a lack of internal expertise may place a restrictive financial burden on these organizations, thereby limiting the availability of innovative medical devices for improving patient quality of life, thus accelerating the removal of these products from the market [24].

In agreement with Behan et al. [19] and Malvehy et al. [11], 54% of study respondents report that their company intends to remove devices currently C.E. marked under the directives from the E.U. market. Possibly one of the most challenging requirements detailed in MDR Article 65 [4] relates to the requirement to conduct a clinical investigation for certain high-risk devices, and the generation of the required data is likely to be cost-prohibitive, leading to the decision to remove the device from the E.U. market [31]. Furthermore, even if, under the MDR, a contract is in place with a second manufacturer which permits full and ongoing access to the device’s technical documentation that second manufacturer is likely to belong to a competitor. Thus it is highly unlikely that manufacturers will avail of this pathway. Indeed, in this study, zero respondents report that their company intends to use a contractual agreement with a second manufacturer to demonstrate equivalence.

In relation to new devices, despite the U.S.A. representing the largest global medical device market, many manufacturers have strategically decided to launch new devices in the E.U. ahead of the U.S.A. This decision has been attributed to several reasons, including lower clinical data requirements, faster approval time, and lower costs [28]. However, introducing stricter MDR requirements for clinical evaluation has forced many manufacturers, particularly high-risk devices, to rethink their global Strategy [10, 24]. Indeed, in this study, approx. 42% of respondents in the high-risk device group plan to launch their new devices in the U.S.A. or another market first and pursue MDR CE marking later. The potential impact of this Strategy is a delay in access to new and innovative medical devices for E.U. patients. In addition, some MedTech intelligence sources have reported pediatric cardiology device shortages due to MDR [31].

In answer to this research question, the main challenges faced by manufacturers when generating n MDR-compliant CER are determining the quantity and quality of clinical data required to generate sufficient clinical evidence and understanding the expectations of the MDR. Due to these challenges, the E.U. market is becoming less attractive for medical device manufacturers [17].

### Research Question 2

What are the main data sources employed by medical device manufacturers to collect information on the use of their devices?

As of June 2022, MDCG has released 11 publications specifically addressing clinical evaluation. Similar to the FDAs guidance documents, these documents, while not legally binding, provide insight into the current thinking and expectations for conducting a clinical evaluation and expected compliance. Therefore, it was unsurprising that in this study, most survey respondents use MDCG guidance documents to stay informed. In addition, more than 80% of respondents remain current via notified body webinars, events, publications, etc. This finding highlights the important role notified bodies played in knowledge transfer and thought leadership in the medical device sector while being mindful of the restrictions imposed on them by European Medicines Agency [32, Sect. 1.2.3].

This study’s findings indicate that companies manufacturing high-risk devices rely more on internal knowledge transfer than external training. Whereas for the companies that manufacture medium-risk devices, a reverse trend was observed with a greater reliance on external training courses to increase personnel knowledge. One possible explanation could be that while the MDR introduces stricter requirements for all risk classifications, the requirements are particularly enhanced for high-risk devices. This may have encouraged high-risk device manufacturers to initially rely on external training following the release of the MDR to enable early planning for and commencement of the transition process. Whereas for medium-risk device manufacturers, the transition to the MDR may lag, leading to increased attendance at external training courses to supplement the team’s knowledge. Additionally, if the manufacturers of high-risk devices also place their devices on the U.S. market, they have experience with the FDA PMA approval process. They would therefore be more familiar with the expectations for generating robust and scientifically valid clinical evidence.

Generating a MDR-compliant CER relies on collecting, evaluating, and assessing a sufficient quantity of high-quality clinical data. For legacy devices, the strength of the clinical data varies considerably, meaning that some clinical data sources are more suitable for demonstrating MDR compliance than others [6]. The findings from this study indicate that irrespective of device classification, supporting clinical data for legacy devices is primarily sourced from the manufacturer’s PMS system, including PMCF activities and scientific literature reviews. In recognition of the stricter requirements for generating regulatory-acceptable clinical data, 50% of medium-risk devices and 60% of high-risk device manufacturers indicate their intention to initiate a new clinical investigation supporting C.E. marking. This may be due to changes in the intended use or design of the device throughout its lifetime, lack of/ineffective PMCF activities, and/or reliance on demonstrating equivalence for initial C.E. marking. Whatever the reason, the need to conduct a new clinical investigation is driven by a significant gap in clinical data and places additional time and financial pressures on the manufacturer [17].

Complaints and analysis of vigilance data (serious incidents, FSCAs, FSNs) represent the most widely used sources of reactive PMS data. However, while these data sources are useful for the early detection of safety and performance signals, their use as a source of clinical evidence is limited, as evidenced by the low ranking assigned to the quality of the collected data.

#### Scientific Literature Reviews and other sources

Scientific literature reviews were identified as the third most commonly used source of reactive PMS data. According to MDCG 2020-6, data derived from this source are generally regarded as an acceptable source of clinical evidence for legacy devices, provided the resulting data are of sufficient quantity and quality (MDCG, 2020). MEDDEV 2.7/1 Rev 4 provides guidance on the conduct of a scientific literature review and is encouraging to note that in this study, > 70% of respondents use this document.

#### PSUR Review

Given that the PMS requirements, including the generation of a PSUR, have been applicable since May 2021 and notified bodies are responsible for verifying that the manufacturer has implemented the required processes during surveillance audits [15], it was surprising that approx. 50% of respondents from high-risk and medium-risk device groups did not identify the review of a PSUR as a data source. One possible explanation could be that at the time of data collection, while all respondents may have established the process for generation of the PSURs, only those manufacturers who have obtained C.E. marking under the MDR prior to May 2021 are actively conducting the annual (class IIb and III) or biennial (class IIa) review and update of their PSURs. For other manufacturers, the focus may be gathering the required clinical evidence and preparing their regulatory submissions to support C.E. marking.

The recent vote by the European Parliament to extend the MDR transition period in February 2023 due to concerns about device supply, notified body capacity and manufacturer preparedness reinforce the findings and concerns raised in this study [33]. In a 537-3 vote, with 24 abstentions, the European Parliament adopted a proposal by the European Commission to delay the transition period due to the aforementioned concerns.

## Conclusion

There is a dearth of literature related specifically to the MDR requirements for increased clinical evaluation. While many papers referred to the Clinical evaluation requirements, there was no in-depth analysis.

The new MDR and the requirement for increased clinical evaluation will ensure a safer device for customers and patients. However, manufacturers will face challenges completing clinical evaluations and gathering data for their CER reports. Coupled with a backlog in N.B.’s capacity to certify to the new MDR, there will likely be supply chain shortages related to European devices. Manufacturers will also likely carry out clinical trials in the U.S.A., which is seen as a more manufacturing and innovation-centered regulatory environment. The announcement of the E.U. Health Commissioner to introduce an amendment to allow manufacturers and N.B.’s have more time to certify devices will alleviate concerns about device supply shortages but validates the aforementioned concerns.

A limitation of the study is the lack of published literature related to clinical evaluation under the MDR. Future research opportunities would be to perform more qualitative studies within the device industry and among the compliance stakeholders to understand the challenges to clinical evaluation under the MDR in more detail. However, under the new E.U. regulations, this study is the first to investigate clinical evaluation in medical devices. This study will be a valuable source of information to academic teaching and understanding regulatory compliance as well as to industry stakeholders to understand the requirements of the clinical evaluation process under the new MDR.

## Data Availability

Data is available on request from the authors.
